# Preconception Health Attitudes and Behaviours of Women: A Qualitative Investigation

**DOI:** 10.3390/nu11071490

**Published:** 2019-06-29

**Authors:** Nadia N. Khan, Jacqueline A. Boyle, Adina Y. Lang, Cheryce L. Harrison

**Affiliations:** Monash Centre for Health Research and Implementation, School of Public Health and Preventive Medicine, Monash University, Clayton VIC 3168, Australia

**Keywords:** preconception, pre-pregnancy, pre-natal, attitudes, health behaviour, pregnancy planning

## Abstract

The preconception period is a critical window in which maternal health can profoundly affect both individual and intergenerational health. Despite its importance, little information about women’s preconception health attitudes, behaviours and information preferences exists, yet these details are vital to inform targeted health communication. Semi-structured interviews were conducted to explore women’s attitudes to preconception health (areas of importance, support sources, enablers and barriers), behaviours (information seeking and health actions taken) and information preferences. Interviews were transcribed, coded and thematically analysed. Fifteen women participated (*n* = 7 preconception, *n* = 7 pregnant and *n* = 1 postpartum). Women perceived optimising lifestyle behaviours including a healthy diet, regular physical activity, reducing alcohol intake and pre-pregnancy vitamin supplementation as important preconception health actions to adopt. Few women acknowledged the importance of formal preconception health checks and screening with health professionals. Barriers to achieving health behaviour change included anxiety, stress and challenges obtaining reputable information. Participants reported a lack of preconception information about supplementation requirements, safe foods and exercise recommendations. Information preferences included the internet or their general practitioner. Whilst women predominantly prioritised optimising diet and physical activity prior to pregnancy, there appeared to be limited awareness of preconception health checks and screening, highlighting a need for broader awareness of overall preconception health and wellbeing.

## 1. Introduction

Health prior to conception is recognised as a critical window with profound and lasting effects across the reproductive life course, impacting on fertility, outcomes during pregnancy as well as short and long term health implications for both women and future generations [1]. The significance of this period has been recognised globally by the World Health Organisation Commission on Ending Childhood Obesity, who have identified preconception as one of six key recommended time-points to prioritise for future childhood and adolescent obesity prevention [2]. 

In the absence of clinical practice guidelines for preconception healthcare in Australia [3], recommendations for primary care by The Royal Australian College of General Practitioners (RACGP) include implementing preconception care for all women of reproductive age (15 to 49 years). This encompasses evaluation of medical history (i.e., reproductive life plan; medical, reproductive and family history; medication use; general physical assessment and vaccination history) and health related behaviours (i.e., folic acid and iodine supplementation; healthy weight; nutrition and exercise; psychosocial health; smoking, alcohol and illegal drug cessation and healthy environments) [4]. These recommendations align internationally, with similar practice recommendations published in the US [5] and UK [6]. Whilst high quality evidence of the effectiveness of preconception interventions on pregnancy outcomes is limited [7], previous research demonstrates intervening during preconception optimises behaviour change including dietary change, folic acid supplementation, smoking reduction, weight loss and control of diabetes, and these may improve pregnancy outcomes including reduced risk of miscarriage, neural tube defects, stillbirth, abnormal birth weight and preterm birth [8,9,10,11].

Despite the importance of this period, women planning a pregnancy are a diverse and difficult to reach population, with little regular healthcare engagement prior to pregnancy and limited awareness of preconception health messages [11,12]. Previous research also demonstrates that even when receptiveness to preconception health information is increased, many women do not perceive themselves to be a high-risk population in need of preconception care [12]. Yet, young women of reproductive age present the population group at highest risk of obesity development with rapid weight gain, suboptimal lifestyle behaviours and related major reproductive, metabolic and psychological complications [13]. Within high and middle income countries, over 50% of women enter pregnancy overweight or obese [14,15,16], with a higher body mass index (BMI) prior to conception increasing the risk of complications during pregnancy including gestational diabetes mellitus (GDM), preeclampsia, caesarean section and the birth of a large-for-gestational-age infant [17,18]. Further, children born to obese mothers are twice as likely to develop childhood obesity, independent of maternal age, race, parity, education, gestational weight gain, child gender and birthweight [19]. 

To better facilitate effective engagement strategies and targeted interventions during preconception, improved understanding of women’s attitudes, behaviours and information needs is warranted. To date, there are few in-depth exploratory studies of preconception health engagement and associated perceived barriers and enablers. To address this, we conducted a qualitative study exploring women’s attitudes, behaviours and information needs during the preconception period in relation to preventive strategies recommended by the RACGP to identify gaps and inform future preconception health initiatives in this setting.

## 2. Materials and Methods

### 2.1. Study Design

A phenomenological qualitative approach was employed, using a constructivist paradigm. This approach was chosen as it allows for an in-depth exploration of women’s attitudes and lived experiences, whilst setting aside any biases and preconceived notions [20]. To fully understand women’s attitudes and behaviours around preconception health, semi-structured interviews, with open-ended questions, were applied. The methods of this study have been reported in accordance with the Standards for Reporting Qualitative Research (SRQR) [21]. This project was approved by the Monash University Human Research Ethics Committee (project 11203). 

### 2.2. Participants, Recruitment and Setting

This qualitative research included participants that expressed interest from a larger pool of preconception women recruited for broader research. The broader research program was conducted across Australia in partnership with an Australian private healthcare insurance provider, Medibank Private Limited. Women who had recently joined or upgraded their private healthcare insurance to include obstetrics cover were invited by Medibank Private Limited to participate in a nation-wide questionnaire. Recruited women from the broader pool (*n* = 418) could express interest in the current study involving a thirty-minute semi-structured interview either face-to-face or via telephone, WhatsApp video call or Face time (both of which allow secure end-to-end encryption). Women were eligible to participate if they were either planning a pregnancy in the next twelve months (preconception), currently pregnant or had given birth in the past twelve months (postpartum); were at least 18 years of age; able to communicate in English and able to perform an interview during the study period (March to August, 2018). In addition to purposive sampling, we employed a snowball recruitment approach, whereby participating women were asked to share the details of our study with friends or family to reach a greater number of women. All women were provided with written and verbal study information, screened for eligibility and asked to provide written informed consent prior to participation. 

### 2.3. Data Collection 

The interview guide (Supplemental File S1) was developed by a member of the research team (AYL) in consultation with a study community advisory group, including community members and representatives from community-based organisations and health services. Demographic details collected from each woman included date and country of birth, pregnancy status, previous births, employment status, education, marital status and partner’s age and employment status. Interview questions captured women’s general attitudes toward preconception health and what health actions they were taking or planning to take in the lead up to pregnancy (i.e., supplementation use, dietary changes, physical activity, weight maintenance, alcohol consumption and smoking habits, preconception information received or sought and any challenges related to achieving optimal preconception health or pregnancy planning) as well as preferred avenue and timing for information provision. We also explored women’s social support and the respective roles of partner, friends and family in influencing attitudes towards preconception health behaviours. Question phrasing was adapted for interviews with women across the context of preconception, pregnancy and postpartum.

Interviews were facilitated by an experienced qualitative researcher (NNK), audio recorded, de-identified and transcribed verbatim by an external transcription agency (Digital Transcripts, Melbourne). Data collection and analysis occurred iteratively and interviews were continued until data saturation was reached and no new themes emerged [22]. 

### 2.4. Thematic Analysis

All interview transcripts were thematically analysed by NNK, using NVivo Version 11 software (QSR International Pty Ltd. Melbourne, Australia. Initially, open coding was performed deductively to code for ideas identified from existing literature and research questions. Emerging codes also arose inductively during this process. Similar codes were subsequently clustered into categories and further unified to form broader themes [23]. In order to enhance trustworthiness and credibility of the analysis, analyst triangulation was employed [24], with 20% of transcripts randomly selected and analysed by an independent researcher (JJ) not involved in data collection ensuring consistency of interpretation. The independent audit trails in NVivo were compared and discussed between researchers in order to identify similarities and discrepancies. Discrepancies in coding were discussed and resolved by modifying or including new codes. Themes were further verified with a member of the research team (CLH) with expertise in preconception health. 

Women’s demographic characteristics are presented in Table 1 and are expressed as frequencies and percentages. Age is presented as median (IQR).

## 3. Results

Twelve women expressed interest in participating in interviews, ten of whom were recruited, with an additional five women recruited by snowballing techniques at which time data saturation was achieved (total of *n* = 15 women recruited). At the time of the interview, seven were planning a pregnancy, seven were pregnant and one had given birth in the past twelve months. The median (interquartile range, IQR) age of women was 33 (IQR: 36–31) years and most (*n* = 11) had not previously given birth. Thirteen women were Australian-born and twelve were employed full-time. All women completed post-secondary education and were either married or in a de-facto relationship. The median (IQR) age of partner’s was 36 (IQR: 37.5–31.5) years and most partners (*n* = 14) were employed full time (Table 1 and Supplemental File S2). Ten women participated in an individual telephone interview and five participated in an individual video interview. 

Five overarching themes arose from the interview analysis: women’s attitudes to preconception health, behaviours and actions during preconception, partner and community influences and support, challenges experienced in preconception and information preferences. 

### 3.1. Attitudes to Preconception Health 

The majority of women felt that preconception was an important time to ensure good health, with regular engagement in physical activity being regarded as one of the most important priorities. Maintaining a healthy diet and taking pre-pregnancy supplements, including folic acid, iodine, iron and vitamin D, were also considered important preconception behaviours by the majority of women. 

“Basically, healthy lifestyle things like exercise and having a base level of fitness and um, and healthy food. Those things were pretty important to me.”—(P11, pregnant, 37 years old, one child).

“I think for me it was really important, I liked going on the multivitamins at you know four months beforehand, because then I’d know that my levels were all worked out and things like that…Because I would hate for something to go wrong in pregnancy and think, oh, what if I could have done something to change that?”—(P3, pregnant, 30 years old, no children).

However, some women were predominantly concerned with their fertility and ability to conceive and overall pre-pregnancy health was not a priority. 

“…healthy pregnancy has not even really occurred to me at this point. I am just like trying to get pregnant.”—(P7, preconception, 33 years old, no children).

### 3.2. Behaviours and Actions during Preconception

#### 3.2.1. Discussing Pregnancy Intention with a General Practitioner

Eleven women consulted their general practitioner (GP) during the preconception period to discuss pregnancy planning. Of these eleven women, six recalled having a blood test done, two underwent genetic disease screening, one had a pap smear and two had their immunisation status checked. 

“Um I have gone to my GP and had a chat to my GP about anything else I needed to do, and she just did some blood tests and vaccination tests and those kind of things.”—(P2, preconception, 33 years old, no children).

“So I went to see my GP probably about six months …. I was not going to say I want to fall pregnant. I was just going to say what should I be doing to give myself and my partner the best chances of falling pregnant and her advice was exactly what I have done. Eat well, lose a bit of weight, um, take the Elevit (i.e., pre-pregnancy multivitamin) at least three months in advance, um, things like that which I, you know, put into practice pretty soon after I had that advice.”—(P9, preconception, 28 years old, no children).

A few women who had signalled pregnancy intention to their GP recalled being dissatisfied with the level of support and information provided. These women felt that their GP did not provide the full spectrum of information, were dismissive or were unaware of what information to provide. 

“I brought up the topic because I’d requested all these screenings and I said, you know, we want to start trying to have a family… I also asked if there is anything else we needed to do sort of in preparation. Um and was just sort of told, no, you should be fine, just come back in six months if nothing happens…I probably would have expected more information from the GP as a sort of trusted source of information. Because I actually made the effort to go in in person and get these tests done and what-not, so it would have been nice to have some sort of further information and guidance from the GP.”—(P1, pregnant, 38 years old, no children).

Three women, all of whom were pregnant at the time of the interview, did not discuss pregnancy intent with their GP and cited reasons including not feeling the need to consult their GP or not having a regular GP to engage with. 

“I could have gone to my GP beforehand and maybe mentioned to her that we were trying and things like that, or that I was planning to try. Maybe she could have added some things in. But um I guess I felt like I did not need it at that point in time, and I think it was more of the thing like, oh, we will just go off contraception, we will see what happens, if we have any problems or it is taking a while then I would go to the GP. But I knew that yeah, that I was, I guess I did not have any issues or concerns, I did not go to her.”—(P3, pregnant, 30 years old, no children).

The remaining woman planned to consult their primary health care provider three months prior to actively trying to conceive.

#### 3.2.2. Information Seeking

In addition to their GP, most women consulted or planned to consult online resources, particularly “Google”, for preconception health information. 

“If I think I need to get some general information, I just search on the internet. Not a particular website, but sometimes if I find some particular article informative, I will just read it and incorporate it.”—(P10, preconception, 32 years old, one child).

Other sources of preconception health information commonly mentioned included physiotherapists, fertility specialist, mobile phone apps, one’s own previous pregnancy experience (for multiparous women) and experiences of family and friends.

#### 3.2.3. Behaviour Change and Lifestyle Modification

During the preconception period, behaviour changes adopted by most women included taking pre-pregnancy supplements and reducing alcohol intake. 

“I knew about … taking folate, having good iron stores, um, and a little bit about iodine in pregnancy, so I took a pre-pregnancy vitamin to make sure I got all of those.”—(P5, postpartum, 37 years old, one child).

“I limited the amount of alcohol that I was drinking. I was, I would have one drink a week, as opposed to three or four, which I was having prior.”—(P12, pregnant, 34 years old, no children).

Most women described themselves as having a healthy lifestyle prior to planning a pregnancy and; therefore, carried forward their previous physical activity routine and dietary pattern into preconception. 

“Just in general I find I feel that I am a pretty healthy person with regards to diet and exercise anyway. So, I did not really have to make too many changes before I fell pregnant.”—(P5, postpartum, 37 years old, one child).

Many women described engaging in physical activity, usually undertaken three days per week, encompassing a range of activities including walking, jogging, swimming, Pilates, CrossFit and/or cardio and strength training. Women generally described their diet during preconception as “healthy”, “balanced”, “nutritious” and “non-restrictive”. Some women either completely eliminated or reduced their caffeine intake. 

“I started drinking lots of water… I do not eat a lot of junk food anymore, really occasionally. I focus on eating a balanced diet, mainly including veggies, fruit and healthy snacks rather than eating chocolates and sweets.”—(P10, preconception, 32 years old, one child).

Stress reduction and weight management (including weight maintenance and weight loss) were also incorporated by a few women during the preconception period. 

When asked if, in hindsight, they would do anything differently during the preconception period, half of all pregnant women and the postpartum woman mentioned that they would not change anything whilst the other half mentioned consulting their GP and addressing some lifestyle issues. 

### 3.3. Partner and Community Influences and Support

Most women kept their pregnancy intent private between their partner and themselves and rarely sought support from family and friends during the preconception period. 

“Um I guess we have not really talked a lot about it to family and friends, we have been a bit vague, we have not been telling people that we are going to start trying, so just in case it takes a while and those kind of things.”—(P2, preconception, 33 years old, no children).

However, some noted learning from the experiences of friends and family members who had previously given birth. 

“A lot of my friends do have kids and things like that. So, I guess you hear out of that default what they might have done and things like that, or more so I would probably pick up from that, um times if they had had bad experiences. Like pain and problems during pregnancy and things like that, and, and I would be like right, I do not want to have that, I am going to go into it nice and fit and strong and things like that yeah.”—(P3, pregnant, 30 years old, no children).

Whilst women described their partners as being supportive during the preconception period, majority recalled that their partners made little to no behavioural or lifestyle changes themselves in preparation for pregnancy. This was mainly attributed to the partner’s perception that they already had a relatively healthy lifestyle prior to preconception. 

“He is um, so supportive…I do not think he would have done anything—like he would have changed anything for himself. I think he would have just gone along with whatever I said really. [laughs] I do not think he would have driven any changes…he probably just thought, she has got it under control…he is very healthy”—(P11, pregnant, 37 years old, one child).

However, a few women felt that their partners were simply unaware of what changes needed to be made but would be inclined to take action if necessary. 

“If the advice was that he ought to do it then, yes, I imagine that he would. Um so it is one of those things that he is perfectly capable and prepared to um receive advice. But he may not—I doubt he is aware of anything and I doubt that unless I actually sort of pushed him to go to the doctor, um, that he would find out for himself.”—(P6, preconception, 32 years old, no children).

Of the few partners who adopted changes, these included reducing alcohol consumption, improving diet, undertaking a preconception health check-up, engaging in physical activity, quitting smoking and taking men’s multivitamins. 

### 3.4. Challenges Experienced in Preconception

While some women experienced no particular challenges during the preconception period, others commonly mentioned stress and anxiety as a challenge in the lead up to pregnancy. Stress was attributed to a range of factors including work, conception, financial stability, social stigma around age, relationship status, miscarriage, and fear of the unknown. 

“I am a bit of a stress head. So, I guess a challenge for me is sort of not being too stressy about it and trying to relax and enjoy the whole process and not be too anxious about things or worry about things going wrong. And maybe with good planning and having all the information, that sort of minimises that level of anxiety. But yeah, that is probably the only challenge I have is just being a bit scared of the unknown. And all the changes in your body and being able to manage everything.”—(P13, preconception, 35 years old, no children).

Additionally, many women found it challenging to source reliable and easily available preconception health information online, noting that information was often “conflicting”, “overwhelming” and “hard to find”. 

"I mean, it is easy to Google stuff online but there is so many websites and stuff like that, you do not know what is legit or what is just one random person’s opinion and what is actually from a professional person. All, um, all that kind of stuff. Yeah, so there is—it is very hard to find reliable information I would say, yeah.”—(P15, pregnant, 34 years old, no children).

### 3.5. Information Preferences

#### 3.5.1. Preferred Topics

Women suggested a range of preferred topics for preconception health information. In particular, food safety, the recommended types and intensity of physical activity and which brand of pre-pregnancy supplements should be taken. 

“There is a lot of foods that you cannot eat while pregnant, um, but it probably would have been helpful to have some information on that maybe before pregnancy, just in case you were pregnant and you did not know it yet and you were eating all these wrong foods and stuff. That would have been helpful information to have.”—(P15, pregnant, 34 years old, no children).

“I guess they do say like if you, if you exercise and all that sort of thing, continue doing it. But I guess if you um, like I have, I have done CrossFit stuff before, so that is a lot of weights and what-not. You know, is that still okay to do, or do I need, or would I need to dial it down? Obviously exercise you are doing it to do, but yeah, if, depending on what you do, do you need to drop things down? Like if you play a contact sport, is that still okay to do, or that sort of thing.”—(P4, preconception, 28 years old, no children).

“I think one thing that I searched for was like multivitamins and thinking what is the best one to take, because you hear about like Elevit and brands that are advertised, but how is that better than like when you go to the shop and you stand there and you look, and you are like, oh my gosh. So, information about yeah that and like when is best to start that as well, I think that is important.”—(P3, pregnant, 30 years old, no children).

Additionally, many women felt that it would be helpful to receive a breakdown of what preconception health actions were important to take and when these should be taken. 

“Even like you know a checklist of things you should start, and you know, when you should start. Probably always good to have a healthy diet and lifestyle, but you know when it really is important, you know just think about your drinking and you know when you really do need to start taking supplements, or when it is best to go to your GP and get things checked and all those kind of things like that…like a checklist would be really good, of timings of when you need to be doing things.”—(P2, preconception, 33 years old, no children).

Other topics of interest included men’s pre-pregnancy health, mental health, general health check-up, financial implications of pregnancy (including difference between public and private health care pathways) and changes to expect during pregnancy. 

#### 3.5.2. Preferred Sources

Online was the most preferred avenue for accessing preconception health information, particularly due to ease of accessibility. Women also felt that it was important for any online resources to be endorsed by a professional or government body to increase trustworthiness. This was followed by the GP, information booklets and mobile phone apps. 

“It probably would have been helpful for me like when the doctor took my Implanon out to get a pamphlet explaining sort of a few things and then maybe directing you to a website, um, like an official proper website that you could get more information from and then, you know, hopefully on the website it would have whoever, it would have written articles there, like you know, their qualifications or something so you knew they were legit, not just as I said a random person again giving you their opinion.”—(P15, pregnant, 34 years old, no children).

#### 3.5.3. Preferred Timing 

Most women felt that it would be appropriate to receive preconception health information at least three months before trying to conceive, as this would allow sufficient time to make any necessary behavioural or lifestyle changes. 

“I certainly think three to six months before you start trying would be great…Because I think that is probably the timeframe, really, that you need to start getting your body in order and that sort of thing.”—(P13, preconception, 35 years old, no children).

## 4. Discussion

In our sample of 15 women of reproductive age, women predominantly prioritised the importance of engaging in healthy lifestyle behaviours such as pre-pregnancy vitamin supplementation, nutrition and exercise as important during the preconception period. Most women signalled pregnancy intention to their GP; however, very few recalled having undertaken a comprehensive health check. Anxiety, stress and difficulties accessing reliable information online were particular challenges experienced during the preconception period. Women indicated a preference in receiving preconception health information at least three months prior to conceiving, particularly regarding supplementation, nutrition and exercise, through a range of platforms, predominantly including online and via healthcare providers. 

Prioritising healthy lifestyle behaviours is consistent with previous findings of a qualitative study exploring fertility-related knowledge in which awareness of the impact of adverse lifestyle behaviours such as smoking, drinking alcohol and poor diet on fertility was high amongst respondents [25]. Additionally, a cross-sectional study exploring women’s and men’s beliefs about lifestyle, fertility and pregnancy across 104 countries found that eating a variety of fruits and vegetables and not smoking or drinking alcohol were perceived as the most important lifestyle behaviours to optimise for a healthy pregnancy [26]. Given the rising prevalence of overweight and obesity [15], and the impact of excess weight on maternal and infant outcomes [17,18,27], it is encouraging to see the prioritisation of a healthy lifestyle prior to conception. The prioritisation of a healthy lifestyle may suggest that women view self-regulating behaviours as more achievable and favourable. Additionally, most women in our study perceived having a healthy lifestyle prior to actively trying to conceive and this may explain why most women carried this behaviour into preconception.

Although supplementation use has been considered less important for a healthy pregnancy by a sample of women and men across 104 countries [26], most women in our study took pre-pregnancy supplements and felt that it was an important action to take. This is consistent with findings from the Australian Longitudinal Study on Women’s Health, which reported ~63% of women were taking at least one dietary supplement during preconception [28]. Given the established evidence recommending pre-pregnancy vitamin supplementation, continued effort to raise awareness of the importance of preconception folic acid and iodine is still warranted. 

Most women in our study signalled pregnancy intention to their GP, sought information and adopted positive lifestyle behaviours preconception. Previous research has indicated that active pregnancy planning, by means of consulting a healthcare provider or searching for information, is associated with greater likelihood of engaging with healthy lifestyle behaviours, such as reducing alcohol consumption and improving diet or folic acid consumption [29,30]. Encouragingly, women in our study had also taken steps to join or upgrade their private health insurance to include obstetrics cover in preparation for pregnancy. This may explain the increased likelihood of pregnancy planning and healthcare engagement in this sample. Private healthcare has previously been reported as an enabling factor for engaging preconception care services [31,32]. Further, increased motivation towards healthy lifestyle to optimise individual health and that of their unborn child is likely when pregnancy intention is high with women more receptive to health related information, as shown here, highlighting a critical opportunity for primary health care providers. 

On the other hand, a small group of women in our study actively chose not to signal pregnancy intent to their health care provider and viewed preconception care as necessary to engage with only when experiencing difficulty conceiving. Our findings, along with previous research [12,25], may provide insight into the findings of van der Zee, who reported that whilst women have a positive attitude towards preconception care, many do not view themselves as belonging to the target population in need of preconception care [12]. Women in preconception are a diverse population not necessarily regularly engaged with the healthcare system. This limited healthcare engagement may reduce risk perception and impact on health awareness. Consequently, women may not perceive healthcare engagement to be of top priority preconception unless a fertility problem exists or develops. Additionally, a few women in our study alluded to dissatisfaction with preconception health information provided by their primary care providers indicating that it may not be perceived as helpful.

The Royal Australian College of General Practitioners (RACGP) recommends that preconception care be provided to all women of reproductive age and encompass medical as well as lifestyle related considerations, consistent with international recommendations [5,6]. Medical considerations include reproductive life planning, assessment of reproductive, medical and genetic history, general physical wellbeing, substance use and vaccinations [4]. Our findings suggest that women have a low awareness and recognition of the importance of medical-related preconception considerations. This highlights the need for multi-strategic approaches in both health promotion to improve awareness, supported by appropriate healthcare provision according to guidelines by GPs when engagement does occur. 

Currently, there are no national preconception health care clinical practice guidelines in Australia [3], which may in part explain the variability in preconception healthcare services that women are reportedly receiving. Creation of an evidence-based national guideline, which is supplemented with consumer resources, may assist in increasing both healthcare provider’s and women’s awareness of the full-spectrum of preconception health-related issues. Previously, Australian GPs have suggested checklists for healthcare providers as an enabler for delivering appropriate preconception care [33]. Similarly, women in our study also felt that a checklist for themselves, which lists key preconception health actions and their suggested timing, may be a useful resource, particularly if accessible online or through their GP.

Despite our sample of women being highly educated and motivated to optimise their preconception health, many experienced difficulties accessing reliable preconception health information and reported confusion over conflicting information. Therefore, whilst the internet may enhance accessibility of information, it may preclude behaviour change, information seeking behaviour and confidence in health knowledge. Importantly, these findings may be exacerbated in more vulnerable populations, including those of low sociodemographic status, of lower health literacy as well as culturally and linguistically diverse populations, emphasising the importance of provision of simple, consistent evidence-based information with broad applicability. 

Preconception health behaviours prioritised by women in this study, such as taking pre-pregnancy supplementation and reducing alcohol consumption, were also identified by a recent scoping review as areas that have consistently been the focus of research studies investigating preconception health [34]. Conversely, the scoping review found that the topic of stress has often been neglected in preconception health research [34], and this is an area that we identified as a challenge for women during preconception. Additionally, our findings also warrant the investigation of men’s preconception health attitudes, behaviours and information preferences as women in our sample identified a need for more information regarding men’s preconception health. Our findings support the suggestions of the scoping review, including advocacy for a wider variety of preconception health behaviours, aside from supplementation use and alcohol consumption, being prioritised in research. 

To our knowledge, this is the first in-depth study exploring the attitudes and behaviours of Australian women towards preconception health. Approximately half of the women in our sample were planning pregnancy and able to provide insight into their current experiences. We ensured trustworthiness of our original thematic analysis by having a subset of transcripts recoded independently by a separate member of the research team, who was not involved in the study design, recruitment or data collection processes. 

Limitations include a highly educated cohort, predominantly born in Australia, which may limit generalisability of the results found. It could also be argued that most participating women were highly motivated and invested in their preconception health as they have voluntarily included obstetrics cover as part of their private healthcare. Additionally, given the inclusion of women who were pregnant or had given birth in the past 12 months at the time of recruitment, our findings may be limited by recall bias. Despite a relatively small sample size it was sufficient for data saturation and similar to that used in studies investigating attitudes to preconception health in samples of Italian and Dutch women [11,12]. 

## 5. Conclusions

Overall, whilst women reported optimising lifestyle behaviours during preconception, including pre-pregnancy supplementation use, alcohol minimisation and healthy diet and physical activity patterns, our findings highlight reduced risk perception and low prioritisation of comprehensive preconception healthcare engagement for health screening. This may be precluded, in part, by perceived difficulty in accessing reliable and consistent preconception health information, despite being a highly educated population. These barriers may be exacerbated in more vulnerable populations, including those of socioeconomic disadvantage, those of a lower health literacy and culturally and linguistically diverse populations. Our results highlight a need for dissemination of simple, accessible evidence-based preconception health information that is applicable to differing target populations and is underpinned by health systems and health policy mobilisation, via innovative, broad reaching campaigns.

## Figures and Tables

**Table 1:** Demographic details and characteristics of *n* = 15 participating women.

Demographic Details and Characteristics	Median (IQR *) or Count (%)
Median (IQR *) age in years	33 (36–31)
Reproductive status	
Preconception ^#^	7 (46.7)
Pregnancy	7 (46.7)
Postpartum ^†^	1 (6.7)
Country of birth	
Australia	13 (86.7)
Other	2 (13.3)
Language other than English	
Yes	1 (6.7)
No	14 (93.3)
Location of residence	
Major city	12 (80.0)
Inner regional	3 (20.0)
Previous pregnancy	
Yes	5 (33.3)
No	10 (66.7)
Previous births	
Yes	4 (26.7)
No	11 (73.3)
Number of children	
0	11 (73.3)
1	4 (26.7)
Employment status	
Full time paid	12 (80.0)
Part time/casual paid	3 (20.0)
Post-school education	
Certificate	1 (6.7)
Bachelor degree	8 (53.3)
Graduate diploma/graduate certificate	1 (6.7)
Postgraduate degree (Masters/PhD+)	5 (33.3)
Marital status	
Married	11 (73.3)
De-facto	4 (26.7)
Partner’s median (IQR*) age in years	36 (37.5–31.5)
Partner’s employment status	
Full time paid	14 (93.3)
Unemployed	1 (6.7)

Abbreviations: * IQR: Interquartile range. Notes: ^#^ Preconception period included women who, at the time of recruitment, were planning to conceive within the next 12 months. ^†^ Postpartum period included women who, at the time of recruitment, had given birth within the past 12 months.

## Supplementary Materials

The following are available online at https://www.mdpi.com/2072-6643/11/7/1490/s1, File S1: Interview question guide, File S2: Women’s (*n* = 15) demographic information.
