# Supplementary material for: Preconception Health Attitudes and Behaviours of Women: A Qualitative Investigation
Source: Nutrients. 2019 Jun 29;11(7):1490. doi: 10.3390/nu11071490 (PMC6682867; doi:10.3390/nu11071490)
Supplement: Supplementary file 1 [file nutrients-11-01490-s001.zip › Supplemental File 1.docx]

**Interview question guide**

*Two versions of the guiding interview/ focus group questions have been developed in consultation with key stakeholders and community members. One set of questions intended for pregnant women and women who have recently given birth and one modified for women pre-pregnancy. These questions will be adapted as appropriate and refined through and iterative process as interviews/ focus groups take place*

Date: Time of interview: Place:

Interviewer:

Interviewee:

| **Pregnant women/ *women who have recently given birth*** | **Women preconception** |
| --- | --- |
| - What are your thoughts about planning for pregnancy?   - Before you became pregnant did you think about taking any actions/ changing behaviour?   *(e.g. take folic acid or other supplements, change your diet, increase or maintain physical activity, lose weight, quit smoking, stop drinking, stop using contraception)*   - - What did you do to try and improve your health before pregnancy (if anything)?   - What does your partner/ community think about planning for pregnancy?   - What health actions are important to you and your community? - Were you given any information about health actions before your pregnancy?   - From who?   - Did you try and find any information about having a healthy pregnancy? - What would you have done differently? *(if pregnant or had prior pregnancy)* - Where and when would you want to get information about having a healthy pregnancy from? - What challenges have you faced in preparing for pregnancy? - Any further comments | - What are your thoughts about planning for pregnancy?   - Do you ever think about it what actions to take/ change behaviour you might make before you become pregnant?   *(e.g. take folic acid or other supplements, change your diet, increase or maintain physical activity, lose weight, quit smoking, stop drinking, stop using contraception)*   - - What does your partner/ community think about planning for pregnancy?   - What health actions are important to you and your community? - Have you ever been given any information about health actions before pregnancy?   - From who?   - Have you ever tried to find any information about having a healthy pregnancy? - Where and when would you want to get information about having a healthy pregnancy from? - What challenges might you face in preparing for pregnancy? - Any further comments |
