# Supplementary material for: Preconception Health Attitudes and Behaviours of Women: A Qualitative Investigation
Source: Nutrients. 2019 Jun 29;11(7):1490. doi: 10.3390/nu11071490 (PMC6682867; doi:10.3390/nu11071490)
Supplement: Supplementary file 1 [file nutrients-11-01490-s001.zip › Supplemental File 2.docx]

| Participant ID | Age | Reproductive status | Parity | location of residencE* | Employment status | Education | Marital status | Partner’s Age | Partner’s employment status |
| --- | --- | --- | --- | --- | --- | --- | --- | --- | --- |
| 1 | 38 | Pregnant | 0 | Major city | Full time paid work | Postgraduate degree | Defacto | 45 | Full time paid work |
| 2 | 33 | Preconception | 0 | Inner regional | Full time paid work | Bachelor degree | Married | 33 | Full time paid work |
| 3 | 30 | Pregnant | 0 | Major city | Full time paid work | Postgraduate degree | Married | 30 | Full time paid work |
| 4 | 28 | Preconception | 0 | Major city | Full time paid work | Certificate | Defacto | 29 | Full time paid work |
| 5 | 37 | Postpartum | 1 | Major city | Part time/casual paid work | Bachelor degree | Married | 36 | Full time paid work |
| 6 | 32 | Preconception | 0 | Inner regional | Full time paid work | Graduate Diploma | Married | 31 | Full time paid work |
| 7 | 33 | Preconception | 0 | Inner regional | Full time paid work | Bachelor degree | Married | 37 | Full time paid work |
| 8 | 30 | Pregnant | 0 | Major city | Full time paid work | Bachelor degree | Defacto | 31 | Full time paid work |
| 9 | 28 | Preconception | 0 | Major city | Full time paid work | Bachelor degree | Married | 33 | Full time paid work |
| 10 | 32 | Preconception | 1 | Major city | Part time/casual paid work | Postgraduate degree | Married | 37 | Full time paid work |
| 11 | 37 | Pregnant | 1 | Major city | Full time paid work | Postgraduate degree | Married | 43 | Unemployed |
| 12 | 34 | Pregnant | 0 | Major city | Full time paid work | Bachelor degree | Married | 38 | Full time paid work |
| 13 | 35 | Preconception | 0 | Major city | Full time paid work | Postgraduate degree | Married | 36 | Full time paid work |
| 14 | 37 | Pregnant | 1 | Major city | Part time/casual paid work | Bachelor degree | Defacto | 32 | Full time paid work |
| 15 | 34 | Pregnant | 0 | Major city | Full time paid work | Bachelor degree | Married | 39 | Full time paid work |

**Based on Australian Statistical Geography Standard-Remoteness Areas 2016.*
